# Contrasting two versions of the 4-cup 2-item disjunctive syllogism task in great apes

**DOI:** 10.1007/s10071-024-01927-w

**Published:** 2025-01-02

**Authors:** Benjamin Jones, Josep Call

**Affiliations:** School of Psychology and Neuroscience, University of St. Andrews, St. Andrews, KY16 9AJ UK

**Keywords:** Logic, Reasoning, Certainty, Uncertainty, Great apes, Primate.

## Abstract

Chimpanzees excel at inference tasks which require that they search for a single food item from partial information. Yet, when presented with 2-item tasks which test the same inference operation, chimpanzees show a consistent breakdown in performance. Here we test a diverse zoo-housed cohort (*n* = 24) comprising all 4 great ape species under the classic 4-cup 2-item task, previously administered to children and chimpanzees, and a modified task administered to baboons. The aim of this study is to delineate whether the divergent results reported from the literature are taxonomic differences or artefacts of their methodologies, while extending the literature to cover the remaining great ape species. We find that apes adaptively adjust their choice behaviour in both variants of the task, but that they perform better in trials where the information provided rules out a location rather than removes one of the food items. In a second experiment involving those subjects who passed the first, along with a group of naïve subjects, we test whether subjects were able to apply the logical operation selectively by including control trials where the correct response is reversed. Performance in standard trials breaks down with the addition of control trials, meaning that if apes did solve the first experiment logically, they are not capable of applying that logic flexibly. Considering this finding, we conclude that a 4-cup 2-item task may not be a suitable test of logical reasoning in great apes.

**Supplementary Information:**

The online version contains supplementary material available at 10.1007/s10071-024-01927-w.

## Introduction

To understand the significance of advanced reasoning to human existence a reader need only note the detail and enthusiasm with which errors in human reasoning have been documented (Kruger and Savitsky 2004). Yet our capacity for advanced reasoning relies only on the appropriate combination of discrete inferences, new knowledge drawn only from held knowledge. As such, the extent to which these inferential processes dictate decision making in non-human species is a crucial element of comparative psychology and is the subject of intense debate.

In the language of logic, inference takes the form of a syllogism - two statements, or premises, which flow to a natural conclusion. For example, “dogs are mammals, all mammals have fur, therefor dogs have fur”. When one premise contains two possibilities connected by the logical operator *or* it becomes a *disjunctive syllogism* – if either A or B is true, and A is false then B must be true. The disjunctive syllogism can more simply be referred to as inference by exclusion.

Inference by exclusion can be tested in the absence of language using a visual search task, choice behaviour in line with rational thought can be considered evidence of logical reasoning. To illustrate this Call’s (2004) 2-cup 1-item task presents a subject, in his case great apes, with two upturned opaque cups one of which contains a target item. When participants were given non-visual evidence by the experimenter shaking one cup or showing them the empty cup, they were able to infer the location of the target item. This task has been used to illustrate inference by exclusion in non-human primates (Call 2006; De Petrillo and Rosati 2020; Heimbauer et al. 2019; Petit et al. 2015), Passeriformes (Mikolasch et al. 2012), (O’Hara et al. 2015, 2016; Pepperberg et al. 2013), domestic dogs (Erdőhegyi et al. 2007) and children as young as 23 months of age (Mody and Carey 2016).

Two-cup tasks can, however, be solved by using a strategy of ‘*maybe-A maybe-B*’, in which subjects need only mark each option as possible locations for the target item, then by avoiding the cup which they know to be empty. In its simplest format, the subject does not even need to mark either option as possibilities and can respond appropriately by choosing what is remaining after avoiding the empty cup. To explicitly test the ‘avoid the empty cup’ hypothesis, Call (2022) baited a pair of cups behind a barrier while a single cup rested on the table untouched, before revealing one of the possibly baited cups and giving the subject a choice between the remaining two. In trials where the empty cup was removed, subjects showed a preference for the possibly baited cup, and in trials where the baited cup was removed, they were indifferent. Therefore finding no support for this non-inferential strategy and suggesting that apes are in fact capable of using inference to solve these tasks.

However, Leahy and Carey (2020) challenge the notion of full-blown inferential reasoning in non-human animals by arguing that solving these tasks does not even require a full understanding of possibility. They propose that pre-verbal children and non-human animals possess only a minimal model of possibility, with learning the language of possibility being a necessary stage in scaffolding a mature understanding. Leahy and Carey’s thesis is that human infants and primates lack the modal concepts *possible*, *impossible*, and *necessary*, with a minimal agent’s model of possibility relying on a process of making only a single simulation of the state of the world which she will treat as fact. In the context of the inference by exclusion paradigm, a participant is not compelled to treat both options as possibilities. She can instead make a guess and once she receives either confirmatory or conflicting evidence, keep or revise her decision accordingly.

The full- and minimal model of possibility can be delineated from one another by Mody and Carey’s (2016) 4-cup task. The task involves an experimenter separately baiting behind a visual barrier two pairs of cups with one sticker each, such that each cup has a 50% chance of containing a sticker. They then remove one cup, show the child that it’s empty, and give them a choice between the four cups. Three-year-old children^1^, but not younger, pick the *target* cup - the remaining cup within the pair that now certainly contains the sticker - at rates significantly above chance. The authors cite this as evidence that they are able to reason through the disjunctive syllogism: A or B, not A therefore B.

Younger children (Gautam et al. 2021b; Mody and Carey 2016) and chimpanzees (Engelmann et al. 2023; Hanus and Call 2014), instead pick the target cup approximately 50% of the time, which Leahy and Carey (2020) interpret as evidence of them having made concrete guesses as to where the two stickers are, the first of which was incorrect, so they revised it, and then choose randomly between their revised guesses. Similarly, in a 3-cup 2-item task in which one cup represents certainty, chimpanzees and young children only choose it in approximately 50% of the trials(51%: Engelmann et al. 2023; 44%: Hanus and Call 2014; 60%: Leahy et al. 2022). Thus, irrespective of their inferential capacities, chimpanzees and young children appear to not give special status to a certain outcome.

While the original 4-cup task reveals an empty cup to test the inclusive *or*, the sticker must be under A or B, Gautam, Suddendorf and Redshaw (2021b) extended the paradigm to include ‘reveal-baited’ trials to test the exclusive *or* relation, the sticker cannot be under A and also under B. In their procedure, a sock puppet took the first guess and managed to find a sticker on 50% of trials. In these trials the correct choice is to switch to the alternate pair of cups, which children don’t reliably do until five years of age^2^. Crucially the subject will only respond appropriately to the 4-cup task, switching to the alternate pair only on reveal baited trials, if they represent the likelihood of each cup within a pair containing a sticker as being dependent on the contents of the other cup.

In an attempt to make the primate variant of the 4-cup task more closely resemble a natural foraging situation, Ferrigno et al. (2021) gave their subjects two choices, first from the initial 4 cups and then from the remaining 3, to create a natural experiment whereby 50% of trials would be reveal empty and 50% would be reveal baited. This new two-choice task takes the form of a logically directed sequential search, rather than a response to an experimenter manipulation. The authors tested nine zoo-housed olive baboons (*Papio anubis)*, of which 4 passed the pre-tests^3^. As a group, these 4 baboons scored above chance in reveal empty trials, 3 subjects were individually above chance and 2 were above the 50% chance rate as set out by the minimal model of possibility. However, the 3 baboons who passed the reveal empty trials were precisely at chance in the reveal baited trials. This demonstrates that reasoning via the inclusive disjunction in a 4-cup task is not limited to humans while supplementing the developmental evidence proposing that reasoning via the exclusive disjunction is more complex than the inclusive disjunction.

Engelmann et al. (2023) have subsequently tested chimpanzees under the 2-, 3- and standard 4-cup tasks, with both the reveal empty and the reveal baited trial-types. Consistent with the literature, they found that a majority of apes passed the 2-cup task but were at 51% in the 3-cup task. However, in contrast to young children (Gautam et al. 2021b) and to the baboons tested by Ferrigno et al. (2021), in the 4-cup task the chimpanzees correctly stayed within the pair on just 48% of reveal empty trials, but fared better on reveal baited trials, correctly switching to the alternate pair on 85% of trials. While the difference between children and chimpanzees is unhindered by the use of the same task, making claims regarding differences between chimpanzees and baboons is problematic because of the differences between the tasks. This is something which Englemann and colleagues (2023) explicitly refrained from doing, as the two studies used different analytical approaches with regard to the expected chance level that they used and the comparisons they made.

To reconcile great apes’ high performance in 2-cup inference by exclusion tasks with their failure in 3- and 4-cup tasks designed to test the disjunctive syllogism, we developed a 2-cup task to explicitly test the disjunctive syllogism (Jones and Call 2024). In it we took a standard 2-cup 1-item search task, but instead of giving chimpanzees information about one of the cups before their choice, we offered them a choice first then revealed the contents of the unchosen cup after. Without revealing their chosen cup, we discarded the unchosen cup along with its contents, and gave the subject the choice between their original choice and a food piece half the volume of the original, placed in a previously unoccupied location. We refer to this task as post-decision wagering because it asks subjects to retroactively evaluate the likelihood of their original choice containing the food piece. We found that subjects adaptively adjusted their rates of taking the half-piece based on the contents of the unchosen cup, selecting it more often when they saw the baited cup removed than when they saw the empty cup removed. This showed that chimpanzees represented the contents of each cup as dependent on one another and which cannot be explained by simply avoiding a shown empty cup.

The current study is motivated by two missing pieces of data from the literature. First, it is unknown how chimpanzees would respond to the two-choice version of the 4-cup task that Ferrigno et al. (2021) used with baboons. One of the reasons that these authors mentioned for designing this task was to make it more similar to a natural foraging task, which might reduce the cognitive load of the original task because subjects directly searching and uncovering the first cup might be more memorable than observing an experimenter uncovering it. Second, it is unknown how great apes other than chimpanzees fare in the 4-cup task. Such data are important to contribute to elucidate the taxonomic distribution of cognitive abilities.

The current research tests the 4 great ape species under both the standard 4-cup task (Mody and Carey 2016; here referred as the one-choice variant), and the 2-choice 4-cup task (Ferrigno et al. 2021; here referred as the two-choice variant). Subjects receive a block of each variant, counterbalanced by order. The two tasks test whether subjects can adaptively switch between pairs based on the contents of the first revealed cup. The difference between these variants is that the information is either from an experimenter’s manipulation or their own directed search. As they are both targeting the same underlying ability, we hypothesise there to be a correlation between performance on the two tasks. In experiment 1 we tested a group of relatively inexperienced great apes under both 4-cup tasks, to ascertain: to what extent the differences in comprehension of the inclusive- and exclusive disjunction between baboons and chimpanzees are artefacts of their respective methods; whether a naïve group will replicate the results of the experienced group tested by Engelmann et al. (2023) in the one-choice variant; and whether non-chimpanzee great apes will perform like chimpanzees in the two conditions of the one-choice variant, i.e., better in the reveal baited than the reveal empty. In experiment 2 we then conduct a follow-up experiment with those subjects who passed the 4-cup task in experiment 1 to rule out the use of a non-inferential win-switch lose-stay strategy. In particular, we presented trials with the standard procedure as in experiment 1 (with one baited cup in each set) and control trials in which we baited two of cups in one set and none in the other. If subjects still switched sets after their first choice was baited in standard trials but not in control trials (and not switched sets after first choice was empty in standard trials but switched in control trials), this would indicate that they were not tied to a fixed rule but applied the reasoning flexibly.

## Experiment 1

### Methods

#### Participants

We tested 24 apes (2 orangutans, *Pongo pygmaeus*, 5 gorillas, *Gorilla gorilla*, 7 chimpanzees, *Pan troglodytes*, and 9 bonobos, *Pan paniscus*) housed at Twycross Zoo, England. Four apes failed the pre-test (1 chimpanzee, 1 bonobo and 2 gorillas) and 1 individual passed the pre-test but only participated in 7 test trials within the available sessions, so her data were not included in the analyses. The sample for experiment 1 comprised 19 apes, 2 orangutans, 3 gorillas, 6 chimpanzees and 8 bonobos (11 female, mean age = 19.5 years). Detailed demographic data and rearing history can be found in Table 1. The apes lived in natural groups and had access to both indoor and outdoor space with vegetation. Water was available ad libitum during testing and the apes received regular feedings of a wide variety of fruits and vegetables throughout the day, and additionally received further enrichment. Testing was completely voluntary and took place within a communal area, conspecifics were free to enter and exit the area during testing, however testing was paused if there was more than one individual at the table. If the experimenter deemed the subject to have been distracted during the baiting or manipulation phase, they reset the trial and repeated it at the end of the session. The apes were largely inexperienced with cognitive testing, our post-decision wagering experiments (Jones & Call, in-prep), which used the same procedure as Jones and Call (2024), were the orangutans’ and gorillas’ first experience with cup-based search tasks, while the chimpanzees and bonobos participated in that task and one other, which tested whether subjects would alter their pointing behaviour in response to an experimenter mistaking their previous choice (Durdevic 2024).

**Table 1:** Demographic details of subjects, age is measured in whole years at the date of their first session.*This subject’s record has no entry regarding his rearing history, but the animal care staff believe him to be mother reared

Species	ID	Sex	Age	Rearing history	Variants
Bonobo	A	Female	26	Parent	Both
	B	Female	12	Unknown	Both
	C	Female	3	Parent	Both
	D	Female	10	Hand	Both
	E	Male	19	Parent	Both
	F	Female	12	Parent	Both
	G	Male	7	Parent	Both
	H	Male	6	Unknown *	Both
Chimpanzee	A	Male	36	Hand	Two-choice
	B	Female	39	Parent	Both
	C	Female	34	Hand	One-choice
	D	Male	18	Parent	Both
	E	Female	15	Parent	Both
	F	Female	32	Hand	One-choice
Gorilla	A	Male	9	Parent	Both
	B	Female	28	Parent	Both
	C	Male	5	Parent	Both
Orangutan	A	Male	33	Parent	Both
	B	Female	5	Parent	Both

#### Apparatus

The experimenter (E) sat opposite the subject with a sliding Table (630 mm x 300 mm) placed between them. Two matching pairs of coloured cups (Ø = 85 mm, height = 130 mm) were used as hiding locations, spaced equidistantly (~ 6 cm) at the front of a sliding table. Cups were arranged in sets, so positions 1 and 2 made up set 1 and were one colour, and positions 3 and 4 made up set 2 and were a different colour. The sets were either red and blue (orangutans and chimpanzees) or pink and yellow (bonobos and gorillas). A U-shaped occluder was used to block the subject’s view of the cups during baiting, which E positioned so that it completely blocked visual access to the pair being baited, while leaving the alternate pair visible (see supplementary material for video examples). Cubes of raw sweet potato (1cm^3^), the highest value food item for all groups (Warren 2023; Appendix A), were used as the target items.

#### Procedure

##### Pre-test

E showed the subject a sweet potato piece by holding it between finger and thumb and, after drawing their attention to it, visibly placed it into one of the two sets of cups, sequentially lifting both cups within the pair in a left-right direction and depositing it under one of them. E then repeated the procedure with the second sweet potato piece and the alternate set. They then lifted one of the cups and moved it to the back of the table behind its original location and, if it had been baited, discarded the food piece into a bucket on the floor, before sliding the table to its forward position so that the subject could reach through the mesh and select one of the three remaining cups by touching it. To pass the pretest subjects had to select a correct cup (chance = 0.5) on 8 from 10 trials within a maximum of 2 sessions^4^.

##### Test

Test trials were split into one-choice and two-choice variants. Subjects received 4 consecutive sessions of each variant, each consisting of 8 trials for a total of 32 trials per variant. Block order was randomised within species, with half of the subjects receiving the one-choice variant first, and half receiving it second. Due to testing being opportunistic and voluntary, 2 chimpanzees only completed the one-choice variant, and 1 chimpanzee only completed the two-choice variant.

The one-choice variant followed the format of Gautam, Suddendorf and Redshaw (2021b), featuring both reveal-empty and reveal-baited trials, but with E removing the first cup in place of the sock puppet. The baiting procedure was the same as the pre-test but the baiting took place behind an occluder and cups started at the back of the board. To start the trial, E lifted the cups and placed them at the front of the board (demonstrating that they were empty), then placed the occluder in front of one pair. E then held the food item above the occluder and waited for the subject’s attention to be drawn to it, before hiding it under one of the two occluded cups, as in the pre-test they lifted each cup sequentially in a left-right direction. E then lifted the occluder and placed it down in front of the second pair of cups and repeated the baiting procedure with a second food item. The order which E baited the pairs was counterbalanced within each session. E then lifted one of the cups and moved it to the back of the board and, and, if baited, discarded its contents, before sliding the table to the subject to choose from the remaining three cups. The two possible trial types were reveal empty, where the removed cup was empty, and reveal baited, where the removed cup had been baited (before E discarded the food item). Subjects received 32 trials per variant (one-choice vs. two-choice), spread equally across the 4 sessions, the location of the food items, the identity of the removed cup (locations 1–4), and its status (baited/empty) were counterbalanced between trials. The left-right orientation of the coloured pairs was alternated between sessions.

The protocol of the two-choice variant followed Ferrigno et al.’s (2021) procedure. The baiting procedure was identical to the one-choice variant, but instead of E removing a cup, the subject was given two choices. If the subject could not detect the location of the target item on their first choice, they should select a baited cup on half of the trials (which they did, see below). For comparison with the one-choice variant, trials where subjects were correct on their first guess will be referred to as reveal baited, and those where they were incorrect will be reveal empty.

#### Coding and data analysis

As the cups were arranged in sets, all analysis is based on the rate of switching between sets, so if cup 3 was removed or chosen in the first guess then choosing either 1 or 2 would be coded as switching, while choosing 4 would not. All sessions were videotaped, and the subjects’ choice was live coded by E. A second coder blind to the purpose of the experiment coded 15% of trials from the video footage, inter-coder reliability was excellent (Cohen’s Kappa = 0.978). All statistics are paired tests unless otherwise stated.

## Results and discussion

**Fig. 1:**
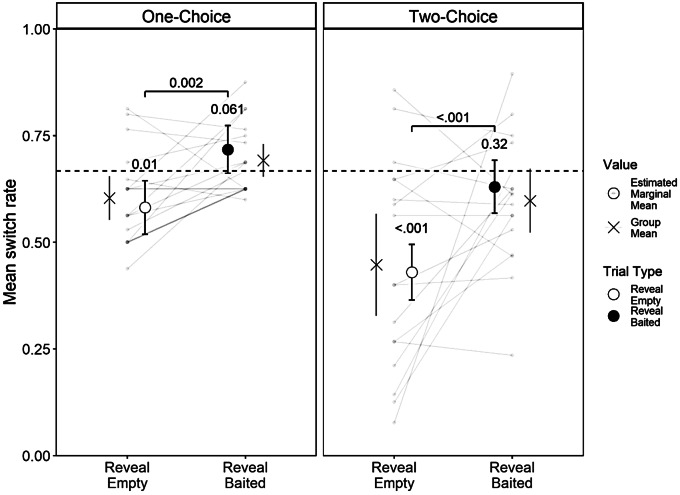
Group and individual level rates of switching pairs by trial type and variant. Points show the estimated marginal means from the model (averaged across species’), along with paired contrasts for switch rates between trial types and z-tests against chance (null = 0.667). The dashed line represents responding at chance, crosses show group means

Figure 1 shows how the rate of switching sets varied as a function of variant and trial-type. To test for differences in the likelihood of switching sets, we fitted a binary logistic regression with a logit link function, using the fixed effects of trial type (reveal empty/ reveal baited), variant (one-choice/two-choice), species and trial number (1–32), and the random effect of ID. As the difference in switch rates between reveal empty and reveal baited trials is our measure of inference, we also included the two-way interactions between revealed cup contents and each of the other predictors. The model with the random effect was not an improvement over a GLM with only the fixed effects (χ^2^ = 2.712, df = 1, *p* = .100), so we continued with the GLM. This model fitted the data better than a model without the interactions (χ^2^ = 17.612, df = 5, *p* = .003), and a null model (χ^2^ = 64.0, df = 11, p = < 0.001). (Coefficients from the final model can be found in Supplementary Table 1.)

We find support for a main effect of revealed cup contents (χ^2^ = 15.646, *p* < .001) and no difference in the size of the effect between the one-choice and two-choice variants (χ^2^ = 0.735, *p* = .391), this shows us that apes can adjust their choice behaviour adaptively in both a self-directed sequential search and in response to an experimenter manipulation. Secondly, we find no support for an interaction between cup-contents and trial number (χ^2^ = 1.891, *p* = .169), so we can reject the conclusion that this is a learned response.

**Fig. 2:**
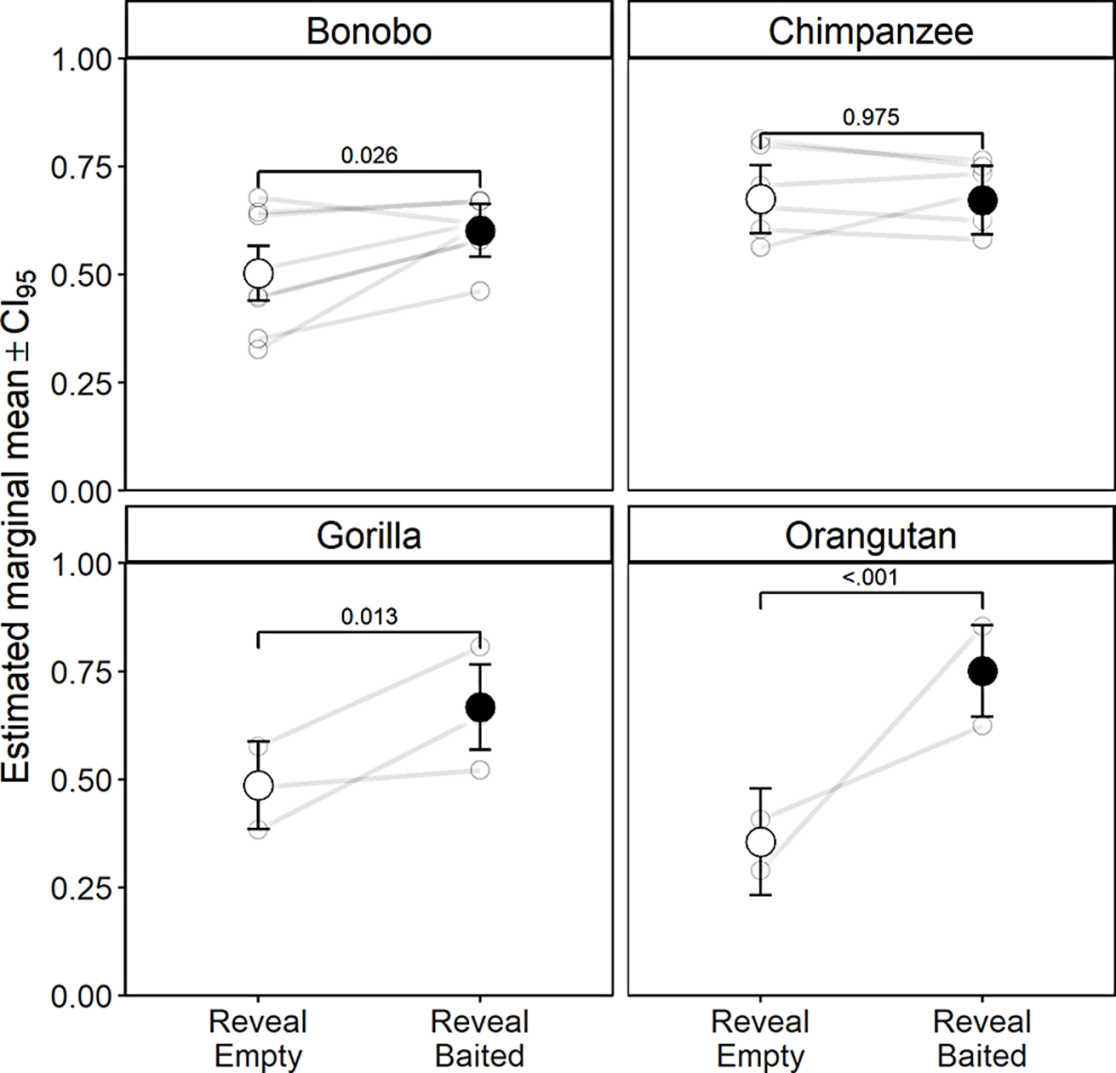
Estimated marginal means from a model to predict switching in Experiment 1 by trial type and species. Light grey points show individual level switch rates

We do report a difference in effect size by species (χ^2^ = 14.39, *p* = .002) (Fig. 2, Supplementary Table 2). Pairwise contrasts reveal a significantly higher rate of incorrectly switching pairs in reveal empty trials for chimpanzees compared to all other species (Supplementary Table 3). The inclusion of the 3-way interaction between trial type, variant and species did not improve the fit of the model to the data (χ^2^ = 9.33, *p* = .156), suggesting these species differences are consistent across variants.

While the chimpanzees did not adapt their switch rate in response to the contents of the revealed cup (β = -0.396, CI_95_ (-1.083, 0.292), this is not necessarily reason to believe that this response is indicative of the species, as similar studies have demonstrated inferential reasoning in chimpanzees under comparable tasks (Call 2022; Engelmann et al. 2023).Instead we could hypothesise that these are effects specific to this group, or to the conditions they were tested under. While in the above cited studies subjects were tested alone in a testing room, we tested subjects in a communal area, so it is possible that social factors played a role in their comparatively poor performance. However, this does not explain why the chimpanzees were the only species impacted by this factor. It is also possible that their performance was impacted by their age, the mean age of the chimpanzees is 29 compared to just 13.5 for the remainder of the cohort. Disentangling the effects of age and species in unbalanced cohort is challenging, however fitting the model with age in place of species, reduces its fit to the data (χ^2^ = 17.98, df = 3, *p* = .001).

We also find a main effect of variant, (χ^2^ = 16.99 *p* < .001), showing that subjects are less likely to switch to the alternate pair in the two-choice task where they engage in a sequential search rather than respond to an experimenter manipulation (Fig. 1). This overall effect of variant on switch rates is relevant because all great ape species have been shown to search under (empty) containers that an experimenter had not visited in an invisible displacement object permanence task but that were located next to other containers that the experimenter did visit (Barth and Call 2006; Mallavarapu et al. 2014). It is possible that the two-choice variant artificially inflates performance on the reveal empty trials (and conversely, primes reduced performance in the reveal baited trials). A hypothetical agent who chose randomly on their first guess and then chose an adjacent cup would only switch on 25% of trials^5^. While this extreme case is not what our data show and does not explain the difference in switch rate between trial types, it does illustrate how a policy of adjacent search could improve performance in reveal empty trials at the expense of reveal baited, highlighting the importance of testing for differences between variants rather than simply absolute rates against chance.

When we compare our model against chance (Fig. 1), subjects switched at a rate lower than chance in reveal empty trials of both variants (one-choice, z-test, z = -2.57, *p* = .010; two-choice, z-test, z = -7.02, *p* < .001) but at chance in reveal baited trials (one-choice, z-test, Z = 1.88, *p* = .061; two-choice z-test, Z = − 0.99, *p* = .320). Which suggests that, like baboons and children older than 2½- but younger 5-years-old, subjects are treating the *or* relation as inclusive (A or B, not A therefore B), but not exclusive (A or B, A therefore not B) (Gautam et al. 2021a). However, this does not mean that apes and children are equivalent in their reasoning because their performance in reveal empty trials is notably worse than that of even the youngest children tested, 2½-year-olds, who only switched pairs on 28% of reveal empty trials (Gautam et al. 2021b).

Our data show a key divergence from a group of chimpanzees tested under the one-choice 4-cup task (Engelmann et al. 2023), who switched pairs in 52% of reveal empty trials, while performing at close to ceiling (85%) in reveal baited trials. In comparison, for the whole cohort we report switch frequencies of 60% and 69% for reveal empty and reveal baited trials, respectively. The conclusion reached by Engelmann and colleagues (2023), and also by Hanus and Call (2014), is that chimpanzees reason probabilistically. Meaning that, without language, chimpanzees do not have access to a concept of certainty so cannot reason ‘logically’ but do however represent the contents of each cup as mutually dependent on each other, so inferentially update their predictions in light of new evidence. Notably, the 52% and 85% switch rates reported for the two trial types (Engelmann et al. 2023) represent approximately equal adaptive divergences away from the chance rate of 66%. If we inspect individual differences in the left pane of Fig. 1, we see a small but consistent effect of revealed cup contents. So in this sense, our cohort level data from the one-choice variant do conform Engelmann’s et al. (2023, see also Call 2022) results, just to a lesser degree, but notably not for the chimpanzees. It may be that this difference is due to differing levels of experience with object search tasks, or cognition research more generally. It is also possible that the difference can be accounted for by Engelmann et al. (2023) only progressing those individuals who had chosen the target cup above chance in the 2-cup task onto the 3- and 4-cup tasks, thus screening out individuals for whom the inference operation was the limiting factor, whereas we simply screened for memory and task comprehension.

**Fig. 3:**
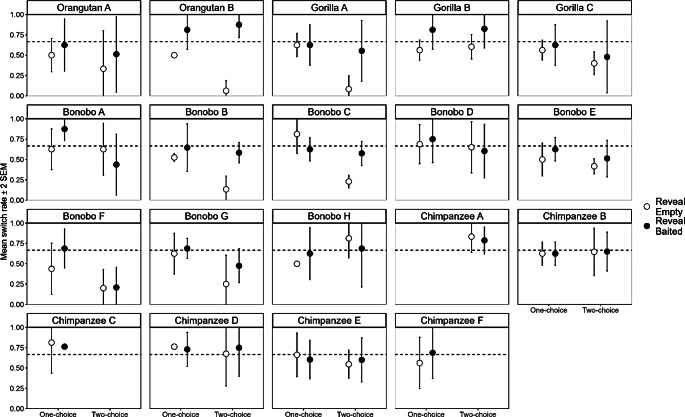
Individual switch rates and standard errors by trial type and variant, arranged by species. The dashed line represents responding at numerical chance p(0.66)

We observed a large amount of individual variation, but the data did not indicate a correlation between performance in the two variants (r_16_ = 0.05, *p* = .841), suggesting that the two tasks are not capturing the same underlying capacity. Figure 3 shows the individual switch rates by variant and trial-type. We used Fisher tests to test for an association between switching and the contents of the revealed cup (Table 2). After accounting for multiple comparisons (Holm-Bonferroni), two individuals showed such a contingency (Orangutan B, *p* < .001, and Gorilla A *p* = .038), but only in the two-choice variant.

Under the minimal model (Leahy and Carey 2020), a minimal agent will switch pairs on 50% of reveal empty trials. When we set chance at this level, we see that both individuals outperform a minimal agent (binomial test, one-tailed, chance = 0.50, Orangutan B: *p* = .002, Gorilla A: *p* = .006), thus we can reject the minimal model as an explanation for their choice behaviour. Additionally, Orangutan B was also above chance in reveal baited trials (binomial test, one-tailed, chance = 0.66, Orangutan B: *p* = .021, Gorilla A: *p* = .385), which suggests that, unlike the baboons tested under the two-choice task (Ferrigno et al. 2021), she was treating the or relation in both its inclusive and exclusive sense.

**Table 2:** Individual switch rates by variant and trial type and the p- value of a fisher’s exact test for differences between trial types adjusted for multiple comparisons using a Holm-Bonferroni correction

		One-choice				Two-choice					
Species	ID	Reveal Empty	Reveal Baited	Fisher Test (p)	Fisher Test (p.adj)		Reveal Empty	Reveal Baited	Fisher Test (p)	Fisher Test (p.adj)	
Bonobo	A	0.625	0.875	0.22	1		0.647	0.467	0.476	0.854	
	B	0.529	0.667	0.491	1		0.125	0.562	0.023	0.127	
	C	0.812	0.625	0.433	1		0.211	0.615	0.03	0.127	
	D	0.688	0.75	1	1		0.6	0.588	1	1	
	E	0.5	0.625	0.722	1		0.4	0.529	0.502	0.854	
	F	0.438	0.688	0.285	1		0.267	0.235	1	1	
	G	0.625	0.688	1	1		0.267	0.471	0.291	0.618	
	H	0.5	0.625	0.722	1		0.857	0.611	0.235	0.618	
Chimpanzee	A						0.812	0.75	1	1	
	B	0.625	0.625	1	1		0.688	0.625	1	1	
	C	0.8	0.765	1	1						
	D	0.765	0.733	1	1		0.647	0.733	0.712	1	
	E	0.647	0.6	1	1		0.562	0.562	1	1	
	F	0.562	0.688	0.716	1						
Gorilla	A	0.625	0.625	1	1		0.143	0.667	0.005	0.038	
	B	0.562	0.812	0.252	1		0.588	0.8	0.265	0.618	
	C	0.562	0.625	1	1		0.4	0.417	1	1	
Orangutan	A	0.5	0.625	0.722	1		0.312	0.625	0.156	0.529	
	B	0.5	0.812	0.135	1		0.077	0.895	< 0.001	< 0.001	

The demonstration that one orangutan can solve both variants of a 4-cup task is an exception within the literature and provides further evidence against language being an pre-requisite for reasoning via the disjunctive syllogism (Jones and Call 2024). However, it may be that this individual has happened upon a non-inferential strategy. If this were the case, we would initially expect to see performance being at chance before abruptly improving and remaining at ceiling for the remainder of the experiment. To test for a learning effect in just those individuals who passed the two-choice variant, we fitted a new GLM to predict the likelihood of responding correctly (switching only on reveal baited trials), using the predictors trial number (1–32) and ID, and their interaction. We found a main effect of ID (χ2 = 16.70, df = 7, *p* = .019), but no effect of trial number (χ2 = 1.31, df = 1, *p* = .251), and no support for an interaction (χ2 = 3.20, df = 7, *p* = .866).

**Fig. 4:**
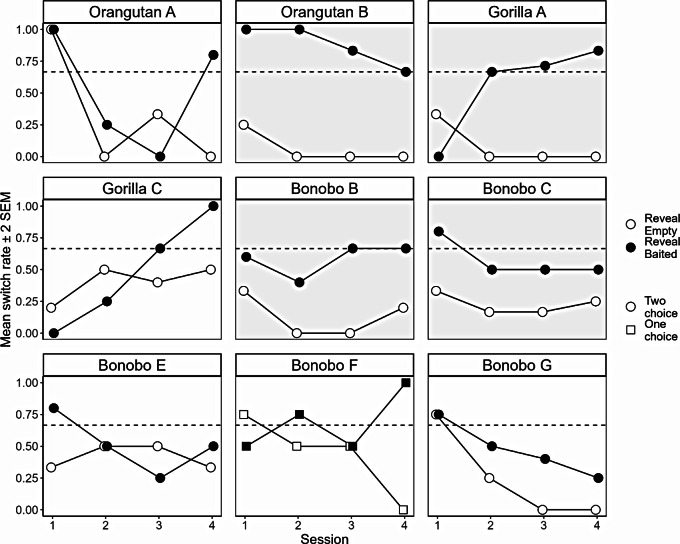
Individual switch rates for reveal empty and reveal baited trials for those individuals who switched at a rate significantly different from chance (binomial test, one-tailed, *p* = .05) in at least one trial type across the 4 sessions of that variant. Shaded boxes show those individuals who switched differentially based on trial-type (Fisher test, two-tailed, uncorrected *p* = .05). The dashed line represents responding at chance

Figure 4 shows how switch rate varied throughout the 4 sessions for each subject who switched at a rate different from chance in at least 1 trial-type (one-tailed binomial test, *p* = .05) (Supplementary Table 4), for all but one individual, Bonobo C, this was the in two-choice variant. If we look at Orangutan B, and to a lesser extent at Bonobos B and C, we see that from the first session they are switching adaptively based on the contents of their first guess, and performance does not markedly improve. Gorilla A, however, shows a profile that is more indicative of associative learning.

One additional consideration is the possibility of observational learning. While testing in a communal area has benefits for welfare, it does have implications for research. Although E paused testing if multiple individuals were seated at the testing area, it was not feasible to do so if they were merely present in the room, nor was it feasible to keep track of which individuals were present during each trial. As a result observational learning remains a possibility. Notably, Bonobo C is the semi-independent offspring of Bonobo B, so her results are a candidate for observational learning.

The failure to detect a learning effect in experiment 1 is evidence against our data reflecting an associative strategy, however if we are to truly justify our conclusion that this is logical reasoning then we must ensure that subjects are able to apply it flexibly and selectively. In experiment 2, we will retest the 9 individuals who scored above chance in at least one variant - trial-type combination, but vary the baiting procedure to include trials where both food pieces are placed into one pair of cups. This was designed to account for a non-inferential strategy of *win-switch lose-stay*, whereby when a subject finds a food piece on their first guess they switch to the alternate set, but if they fail to find a piece, they should stay within the set. This will allow us to delineate those individuals who passed experiment 1 via reasoning logically, who will continue to perform well, from those who deployed this associative strategy, which will now be ineffective on 50% of trials. To assess the potential effect of having participated in experiment 1, we also tested a second group of chimpanzees who were experienced with cup-based search tasks (Jones and Call 2024; Many Primates et al., 2019) but had not participated in experiment 1.

## Experiment 2–4 cup control

### Methods

#### Participants

The 9 individuals who scored higher than expected by chance in at least one variant of experiment 1 progressed to experiment 2 (5 bonobos, 2 gorillas and 2 orangutans). To test whether their participation in the current experiment was affected by the administration of experiment 1, we also tested 9 chimpanzees at the Budongo Research Unit (BRU) in Edinburgh Zoo. These chimpanzees were research experienced but had not participated in experiment 1. Like the Twycross group, subjects are housed in a species typical group with access to indoor and outdoor spaces with vegetation, where testing takes place in a communal area accessible to all individuals during the testing period. The apparatus and procedure were the same but whole grapes were used as target items for the Edinburgh group, as it is their highest valued food item (Warren 2023; Appendix A), while the Twycross group continued to receive cubed sweet potato. To proceed to testing, the Edinburgh chimpanzees needed to score 8 out of 10 on the same memory pre-test task described in experiment 1. Three subjects failed to reach this criterion in two sessions. The final sample comprised 15 apes (7 female, mean age = 18.2), demographic details of all apes can be found in Table 3.

**Table 3:** Demographic details of subjects

Location	Species	ID	Sex	Age	Rearing
Edinburgh	Chimpanzee	G	Female	26	Parent
		H	Female	42	Parent
		I	Female	29	Parent
		J	Male	29	Parent
		K	Male	30	Parent
		L	Male	8	Parent
Twycross	Bonobo	B	Female	13	Unknown
		C	Female	3	Parent
		F	Female	12	Parent
		E	Male	20	Parent
		G	Male	9	Parent
	Gorilla	A	Male	9	Parent
		C	Male	5	Parent
	Orangutan	B	Female	5	Parent
		A	Male	33	Parent

#### Apparatus

The apparatus was the same as experiment 1.

#### Procedure

##### Pre-test

The Edinburgh group performed the pre-test described in experiment 1, the Twycross group did no further pre-test and experiment 2 followed directly from experiment 1.

##### Test

In standard trials the procedure was identical to experiment 1. In control trials both food pieces were placed into one pair of cups, so subjects should choose both members of the baited pair. In control trials, E first showed the subject that all cups were empty and then baited the first pair as before, this time instead of placing the occluder in front of the second pair of cups, they placed it back down in front of the same pair and baited it again, so both cups within the pair contained a food item. This means that the un-baited pair were never covered and never touched by E. Each 12-trial session contained a block of six one-choice trials and a block of six two-choice trials, the order of which was counterbalanced between session. Within each 6-trial block there were four control trials and two standard trials each randomly distributed, meaning that over the three sessions subjects received 12 control trials and 6 standard trials for each variant, for the one-choice variant these were split evenly between reveal-empty trials, where E selected from the unbaited pair, and reveal-baited trials where they removed a baited cup.

##### Data coding and analysis

As in experiment 1, analysis is based on the rate of switching between pairs, as the removed cup was not replaced, chance was set at 66%. Inter-coder reliability for 15% of trials was perfect (Cohen’s Kappa = 1). For analysis of two-choice control trials, error types were coded as follows: *Correct both* - the subject correctly chose both cups from the baited pair; *Incorrect 1st choice* - the subject chose the un-baited pair on their first guess; *Incorrect 2nd choice* – the subject chose correctly on the first choice but switched to the alternate pair on their second choice; *Incorrect both* – the subject chose both cups from the unbaited pair.

## Results and discussion

**Fig. 5:**
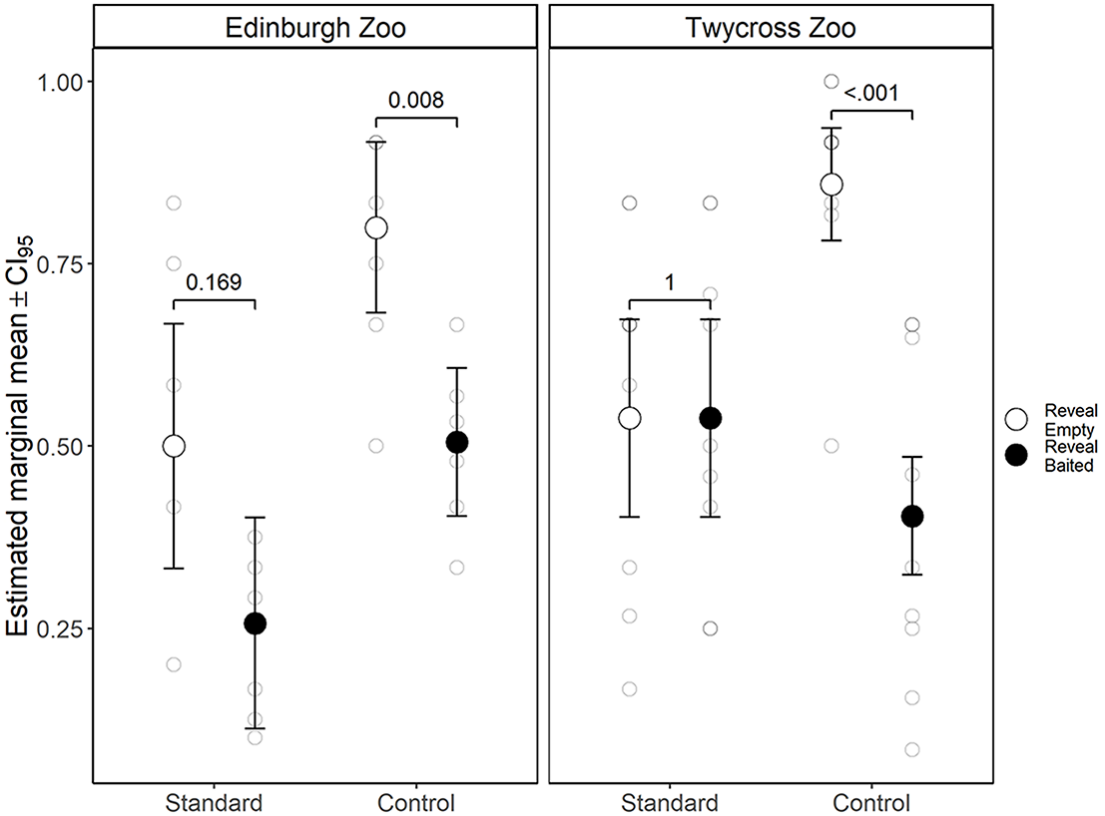
Estimated marginal means for a model to predict switching in experiment 2 by trial type and group. Small circles show switch rates at the individual level. The variant term (one-choice vs. two-choice) was not informative, so it was removed from the model (χ^2^ = 10.40, df = 8, *p* = .238)

Figure 5 shows how switch rates varied by condition. To test for differences in the likelihood of switching pairs, we fitted a mixed effects model with logit link function, using the predictors trial type (reveal empty/reveal baited), variant (one-choice/two-choice), condition (standard/control) and location (Edinburgh/Twycross) along with all interactions and the random effect of ID. The random effect did not improve the fit of the model over a GLM with only the fixed effects (χ^2^ = 1.364, df = 1, *p* = .243), so we continued with the GLM. Similarly, we found no main effect of test variant (one-choice/ two-choice) or support for any interactions containing it, so we removed it, which did not influence the model’s fit (χ^2^ = 10.40, df = 8, *p* = .238). The resulting model, using the predictors trial-type, condition, location and their interactions, fit the data better than a null model without the predictors (χ^2^ = 82.51, df = 15, *p* < .001).

As the baiting procedure was different between control and standard trials, the correct response to each trial type (reveal empty or reveal baited) is reversed between conditions^6^, so the interaction term is more informative than trial type alone. This also serves to avoid perceptual enhancement, which control trials are vulnerable to due to the experimenter only acting on one pair of cups, which would act equally on both trial types but not change the magnitude of the difference between them. We found that the effect of revealed cup contents varied by condition (χ^2^ = 12.77, *p* < .001), which shows that subjects were switching differentially based on the baiting procedure and not simply responding based on a decision rule. However, while pairwise contrasts (Supplementary Table 5) show that both groups switched differentially in the control condition (Edinburgh, β = 1.365, CI_95_ (0.529,2.201), *p* = .008; Twycross, β = 2.195, CI_95_(1,474,2.915), *p* < .001) neither group did so in the standard condition (Edinburgh, β = 1.06, CI_95_ (0.048, -2.07), *p* = .169; Twycross, β = 0, CI_95_ (-0.771, 0.771), *p* = 1). This suggests that the presence of the control trials made the standard trials more challenging for the Twycross group, who switched differentially in experiment 1 (paired t-test, t_8_ = 4.16, *p* = .003)^7^.

Both groups significantly altered their choice rates between conditions in reveal empty trials (Edinburgh, β = 1.386, CI_95_ (0.394, 2.379) *p* = .032; Twycross, β = -0.542, CI_95_ (0.814, 2.492), *p* = .001) but not in reveal baited trials (Edinburgh, β = 1.082, CI_95_ (0.222,1.943), *p* = .065; Twycross, β =-0.542, CI_95_ (-1.183,0.099), *p* = .346). Additionally, the 3-way interaction was significant (χ^2^ = 4.85, *p* = .028), showing that while the responses of the two groups to each condition are comparable, the magnitudes differ.

Particularly interesting is the performance of the Edinburgh group in standard trials, who switched approximately 50% of the time in reveal empty trials, which reproduces the performance of the Ngamba Island chimpanzees tested by Engelmann and colleagues (2023); but in reveal baited trials, subjects correctly switched sets on just ~ 25% of trials, compared to ~ 85% in the Ngamba group, suggesting that the theory which Engelmann et al. (2023) propose to explain their data is not sufficient to describe ours.

The data from both conditions suggest that the presence of control trials, where the correct response was reversed, made the standard trials more difficult. While the variety of test trials presented within a session may have placed large cognitive demands on the subject, the intention of this experiment was to test for the flexible application of inference, which this experiment has failed to find evidence for. This suggests that reasoning via the disjunctive syllogism in a 4-cup 2-item task is outside of the capacity of great apes. This does not categorically disqualify them from reasoning logically but, under this task, apes do not perform at rates better than chance. If we were to alter the presentation of this task, such as by performing a single trial per session and increasing the value of the reward, we may see different results.

### Analysis of errors in two-choice control trials

The original two-choice variant can be solved using a simple decision rule of win-switch lose-stay, whereby if they are unsuccessful on their first guess they stay within the pair, but if they are successful, they switch to the alternate pair. Incorrect deployment of this rule in two-choice control trials would result in the subject being either correct on their first guess but incorrect on their second or, if they were disregarding the baiting, being incorrect on both guesses.

**Fig. 6:**
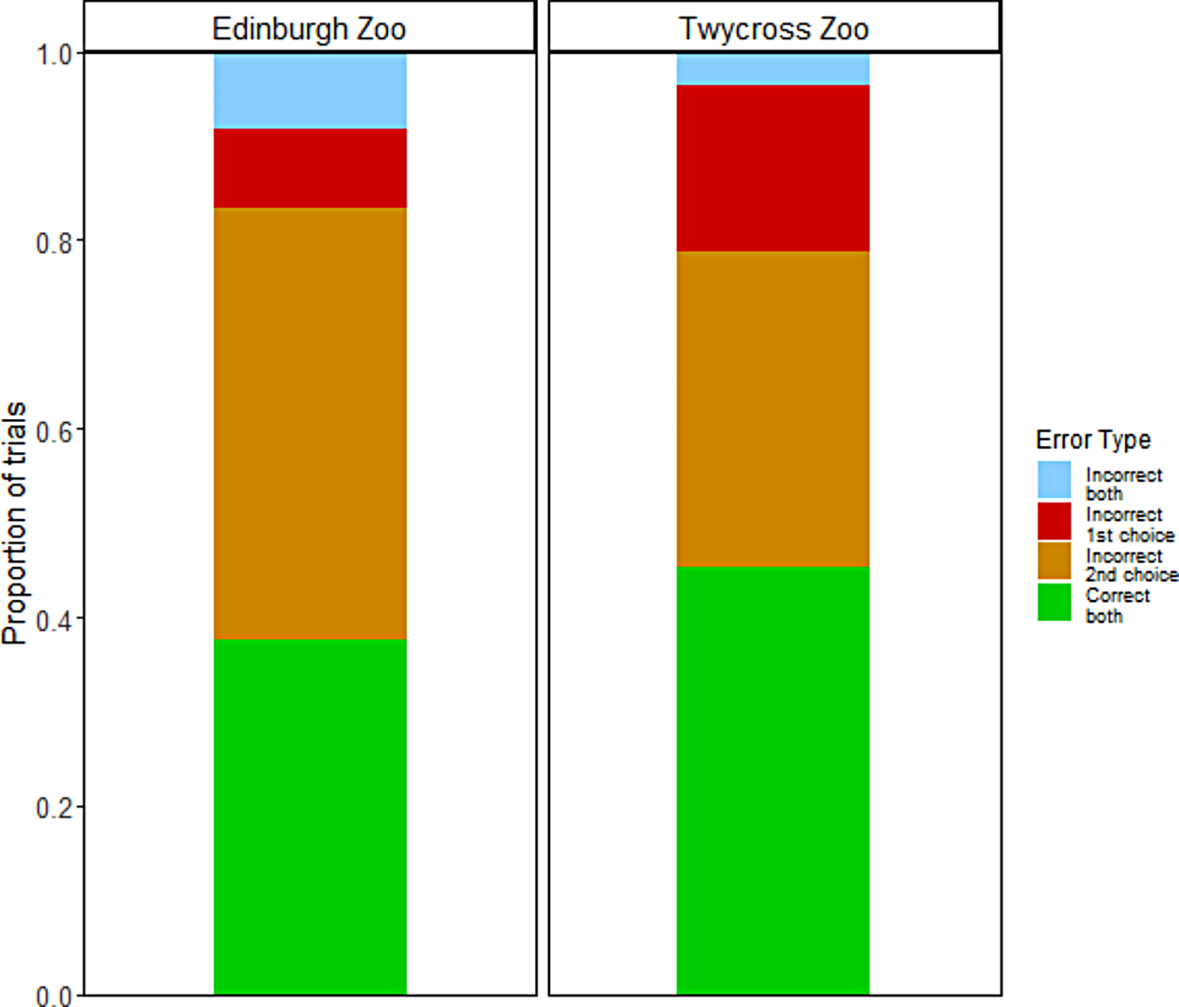
Distribution of error types in two-choice control trials of experiment 2 as a function of group. Incorrect deployment of a win-switch lose-stay strategy would manifest as choosing correctly on the first guess and then incorrectly switching to the alternate pair (orange bar), or choosing incorrectly on the first guess and then staying within the same pair (blue bar)

Figure 6 shows the distribution of errors in two-choice control trials. While the Edinburgh group made win-switch lose-stay errors more frequently (54.2% vs. 37.0%), the overall distribution of error types did not vary between groups (χ^2^ = 6.01, df = 3, *p* = .111), which suggests that, while the error rate is surprisingly high, the Twycross group have not learned this response pattern through reinforcement in Experiment 1.

**Fig. 7:**
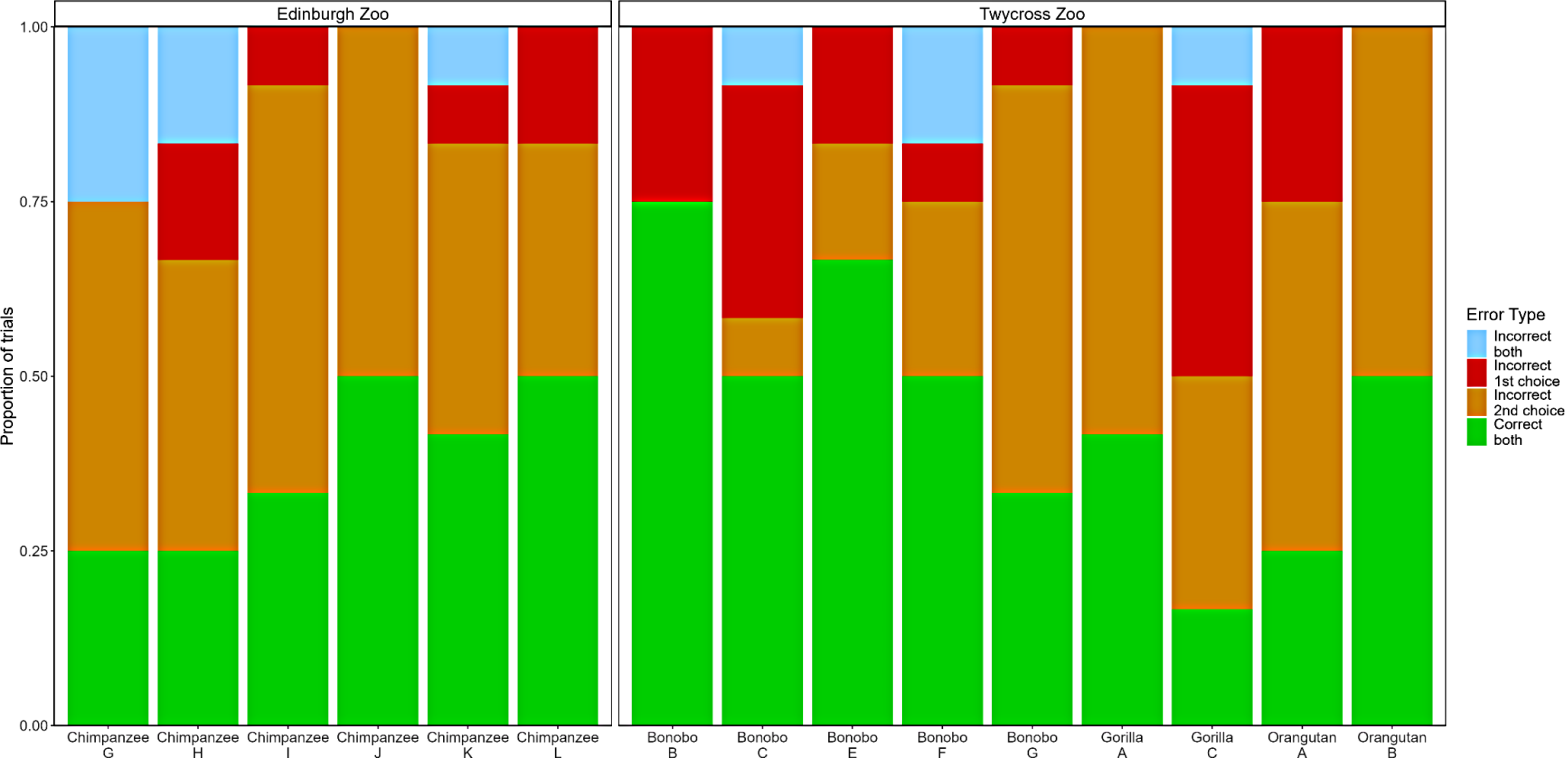
Individual level error distribution in two-choice control trials

Figure 7 shows the error distribution at an individual level in two-choice control trials. We see that Gorilla A and Orangutan B, the individuals who scored highest of the group in the two-choice variant in experiment 1, make some errors on control trials indicative of having used a win-switch lose-stay strategy. Both individuals chose correctly on all of their first guesses, indicating that they were attending to the baiting, so the second guesses could alternatively be a reflection of simply choosing randomly, potentially due to cognitive overload from the varied baiting procedure. Chimpanzee J also demonstrated this pattern of responses, without having participated in Experiment 1. A key limitation of the control trials is that if subjects attend to the baiting process, it becomes impossible to test for the lose-stay strategy, as only the win-switch strategy can be assessed.

Nonetheless, while these errors mean that we must treat the results of experiment 1 with caution, they do not necessarily rule out a non-associative interpretation. These tasks involve a high number of trials, each of which require a large amount of cognitive effort, but in return the reward for each is relatively small. Therefor this task is a viable candidate for the use of a heuristic, a simplified decision rule that serves to offer an approximately accurate answer in exchange for a drastically reduced cognitive load (Rieskamp and Hoffrage 1999; Shah and Oppenheimer 2008). Previous literature has shown that primates also resort to heuristics under tasks with a high cognitive load (Broihanne et al. 2019), so it is plausible that, over the course of the 8 sessions of experiment 1, a cognitive strategy was replaced by a heuristic one, which the apes incorrectly applied in control trials of experiment 2.

The formation of a useful heuristic was possible in the first experiment because the baiting procedure was consistent throughout, but this also meant that failing to attend closely to it was not penalised, which could also have decreased either the cognitive or attentional load for available inferential processes. While the aim of this experiment was to force the subject to attend to the baiting procedure and flexibly apply the underlying logical operation, it is possible that this was the limiting factor in the task and results are due to subjects responding entirely at random. With this regard, it is also significant to look at the ~ 20% of first guesses directed towards the unbaited pair.

Nevertheless, the results of experiment 2 suggest that subjects are not able to solve the 4-cup 2-item task *logically*. Under a standard logic account (Rips 2001) we apply logic sparingly to evaluate focussed parts of our model, so in a standard trial an agent can simply address the pair which is acted on in trials and would continue to perform well despite the addition of the control trials. Similarly, in control trials, the subject could eliminate two locations prior to the first cup being revealed and even a policy of simply avoiding the empty cup would be sufficient to pass the task. We fail to see this, even with stimulus enhancement theoretically biasing an even greater number of second choices towards the correct pair.

### General discussion

Across two experiments we have investigated whether great apes are able to reason logically in line with an understanding of the disjunctive syllogism. Firstly, we found that, as a group, subjects adaptively switch between pairs depending on the contents of the revealed cup in both the one- and the two-choice task. Secondly, we have shown that great apes perform better in trials that test the inclusive than the exclusive disjunction (compared against chance responding). At the individual level, we have shown that one orangutan was able to respond appropriately to both the inclusive and the exclusive disjunction in a 4-cup task, demonstrating that language is not required to reason logically in this task. Using a second experiment involving those subjects who passed the first experiment along with a group of naïve subjects, we showed that their responses are not a learned heuristic, but they were likely based on paying attention to both the baiting and the outcome. However, the addition of control trials resulted in a decrease in performance compared to experiment 1, meaning that apes were not able to flexibly apply the inference needed to solve the standard trials in experiment 2.

Both of these groups of apes have previously passed our 2-cup disjunctive syllogism task (Jones and Call 2024; in-prep), which suggests that performing the inference operation is not a limiting factor in apes’ performance. When we combine this finding with the observation that some subjects are able to pass this task without being able to flexibly apply the same logical operation, it may indicate that the 4-cup task is not a suitable test for reasoning ability in great apes, especially when paired with control trials. Nevertheless, the task highlights a significant distinction between humans and non-human primates, making it an important contribution to the literature. An alternative task that reduces executive demands, may offer a more precise delineation of where this divergence occurs.

The difference in performance between the 2-cup and 4-cup tasks may be due to the working memory demands of tracking two food items across four possible locations. However, working memory limitations cannot explain infants’ failures, as infants can track small sets (Feigenson and Carey 2003) and keep the resulting representations separate (Rosenberg and Feigenson 2013), but still fail the 3-cup (Leahy et al. 2022) and 4-cup tasks (Mody and Carey 2016). While this explanation may not apply to children, this does not rule out its utility, as divergence between apes and 3-year-old children on the 4-cup task already indicates that different elements of the task are the limiting factor in performance for these groups. Expanding this developmental literature to great apes may provide explanations for the root of their failure. Alternatively, as suggested by Krupenye and Call (2019) when discussing why great apes had failed early, food-based theory of mind tasks, the inhibitory and attentional demands which food places on apes may be masking cognitive abilities. While no explanation is sufficient in isolation, it may be that the cognitive demands placed on the subject by multiple elements of the task are contributing to great apes’ failure.

The significance of language to modal logic is comparable of the distinction between inductive and deductive inference, which share the end-goal of using held knowledge to derive new knowledge, but differ from one another is the method by which they reach the new knowledge. Deductive reasoning uses strict rules or axioms, statements which are known to be universally true, to make new prepositions from initial premises; induction, by contrast, does not require formal rules and instead works via drawing logical conclusions based upon previous observations (Henderson, 2020). The characteristic difference being that deductive inference relies on absolute truths, while inductive inference relies upon probabilities. While non-human animals may be capable of tracking environmental regularities, having intuitions, and making rational choices based on them; these conclusions are likely still probabilistic, as deductive reasoning is an entirely language-based concept. Grigoroglou and Ganea (2022) note that children do not start to use the semantic meaning of the modal verbs in an adult sense until the age of 7, so it is plausible that the young children tested by Mody and Carey (2016) and by Gautam, Redshaw and Suddendorf (2021b), are also responding simply with intuitions. While they may not be reasoning deductively, the question remains as to why reasoning via the disjunctive syllogism in a 4-cup task is near-ubiquitous in children by the age of 5 but absent from all but a handful of apes tested.

## Conclusion

Confronted with both the one-choice and the two-choice 4-cup task, great apes switch adaptively in line with reasoning via the disjunctive syllogism. At an individual level, two individuals switched adaptively based on the contents of the revealed cup and their results cannot be explained by the minimal model. Thus, we have shown that passing both the inclusive and the exclusive variants of the disjunctive syllogism in a 4-cup task is not unique to humans. However, when we include associative control trials, which were absent from previous studies, we find that performance breaks down, suggesting that in this setting, apes are not able to apply the underlying logical operation flexibly. When we consider this finding alongside the evidence that chimpanzees can solve a 2-cup disjunctive syllogism task (Jones and Call 2024), we conclude that despite its conceptual sophistication, the 4-cup 2-item disjunctive syllogism task may not the best choice to measure logical reasoning in non-human animals due to its executive function requirements.

## Electronic supplementary material

Below is the link to the electronic supplementary material.

Supplementary Material 1

Supplementary Material 2

Supplementary Material 3

## Data Availability

Data within the manuscript has been anonymised at the request of Twycross zoo, de-anonymised individual data for the purpose of secondary analyses are available upon request. Raw data is provided within the supplementary information files.

## Footnotes

1 Later revised to 2½ by Gautam, Redshaw and Suddendorf (2021b).

2 The minimal model predicts correctly switching in 100% of the reveal-baited condition, when one of the two pieces are removed the subject revises their model with regard to the target cup, and searches for the remaining sticker in the alternate pair.

3 The first pre-test required that subjects pass a 2-location 1-item inference by exclusion task, 5 individuals passed this task. The second ensured that subjects were willing to touch all 4 locations by visibly baiting one location per trial, all 5 passed this. In the final pre-test the experimenter visibly baited 2 locations and gave the subject two guesses, the requirement was not that they choose correctly but that the choose two different locations, 1 subject failed this stage,

4 Chance is set to 0.5 because for 50% of trials a baited cup was removed, therefore one baited cup remained so *p*(Correct) = 0.33 and for 50% an un-baited cup was removed, so two baited cups remained and *p*(Correct) = 0.67.

5 In 50% of first choices, they would choose an *inner* cup (2,3), so would choose within the pair on 50% of trials. In the other 50% of first choices, they would choose an *outer* cup (1,4) on their first guess, so would choose within the pair on 100% of trials. For a resultant switch frequency of 25%.

6 For example, in the standard condition there is one piece in each pair, so the correct response to reveal empty trials is to stay within the set as the revealed cup’s partner certainly contains a piece, but in control trials subjects should switch because both cups in the alternate set were baited.

7 This comparison is based on individual rate is averaged across one-choice and two-choice trials of experiment 1, for the 9 individuals who participated in both experiments.
