# Correction: Contrasting two versions of the 4-cup 2-item disjunctive syllogism task in great apes

**DOI:** 10.1007/s10071-025-01996-5

**Published:** 2025-07-26

**Authors:** Benjamin Jones, Josep Call

**Affiliations:** School of Psychology and Neuroscience, University of St. Andrews, St. Andrews, KY16 9AJ UK

**Correction: Animal Cognition (2024) 28:3**

10.1007/s10071-024-01927-w

In this article the ‘Conflict of Interest’ statement was missing and should have been “Josep Call is an editor for the journal and has not taken part in the manuscript’s peer review process.” The original article has been corrected.

